# Nicotine dependence, motivations, and intention to quit smoking among smoking cessation outpatients: A cross-sectional study

**DOI:** 10.18332/tid/205671

**Published:** 2025-07-18

**Authors:** Lingwei Chen, Zhenbo Tao, Qianqian Xu, Yingying Zhu, Shige Ding, Ying Dong

**Affiliations:** 1 Ningbo Municipal Center for Disease Control and Prevention, Ningbo, China

**Keywords:** intention to quit smoking, nicotine dependence, smoking cessation outpatients, motivation

## Abstract

**INTRODUCTION:**

Intention to quit smoking, a well-established predictor of future cessation attempts, is related to individuals' motivational drivers for quitting. While prior studies have examined nicotine dependence and motivations in predicting quit intention among general smokers, the unique profiles of smoking cessation outpatients in China remain underexplored.

**METHODS:**

This cross-sectional study recruited 703 smokers who visited the standardized smoking cessation clinic at Ningbo, China, between January 2023 and January 2025. Sociodemographic characteristics, Fagerström test for nicotine dependence (FTND), motivations (including health, family, social, and self-management reasons), and intentions to quit smoking were collected by questionnaire-based investigations. Logistic regression analysis was applied to calculate adjusted odds ratios (AORs) and 95% confidence intervals (CIs) for identifying the factors associated with intentions to quit smoking.

**RESULTS:**

In all, 59.60% of outpatients (n=400) planned to quit smoking within the next 7-day period, including those initiating abstinence, and were categorized as the high intention to quit smoking group. FTND scores classified 45.09% (n=317) as mildly dependent, 42.96% (n=302) as moderately dependent, and 11.95% (n=84) as severely dependent on nicotine. The proportion of patients in the high-intention group who quit smoking for health reasons was significantly higher than that in the low-intention group; for instance, the rates of smoking cessation attributed to personal health diagnoses were 28.25% and 4.62%, respectively. Moderate (AOR=2.77; 95% CI: 1.78–4.29) and severe nicotine dependence (AOR=2.53; 95% CI: 1.27–5.04) were independently associated with heightened cessation intention relative to mild dependence among smoking cessation outpatients. Duration of smoking (AOR=0.98; 95% CI: 0.96–1.00), drinking (AOR=0.31; 95% CI: 0.18–0.54), and self-reported health (AOR=1.60; 95% CI: 1.01–2.55) had significant effects on intention.

**CONCLUSIONS:**

The intention to quit smoking was positively associated with the degree of nicotine dependence and health- and family-related motivations. Doctors should take these characteristics into account to provide personalized smoking cessation services.

## INTRODUCTION

The tobacco epidemic poses one of the biggest public health threats the world has ever faced, ranking among the top three contributors to the global disease burden^1^. Tobacco smoke contains over 80 known carcinogens, with substantial epidemiological evidence linked to cardiovascular diseases, respiratory diseases, and at least 20 different types or subtypes of cancer^2,3^. Globally, 1.25 billion people use tobacco, and 80% of them live in low- and middle-income countries (LMICs), where the burden of tobacco-related illness and death is heaviest^4^. China, the world’s largest tobacco market, accounting for approximately 40% of global cigarette consumption, reported >300 million active smokers with male smoking rates >50%^5,6^. Projections indicate that annual tobacco-related deaths in China will exceed 3 million by 2050 without effective interventions^7^.

China signed the WHO Framework Convention on Tobacco Control (FCTC), the inaugural global public health treaty targeting tobacco regulation, in 2003, and it entered into force in the country in 2005. To aggressively control tobacco, the National Health Commission of the People’s Republic of China launched the Healthy China Initiative (2019–2030)^8^. This policy encompassed standardized counseling protocols and improvements in pharmacotherapy accessibility to optimize the delivery of smoking cessation services. There were 366 institutions providing smoking cessation treatment via dedicated clinics in mainland China^9^. Generally, multidisciplinary teams led by pulmonologists, psychiatrists, or general physicians deliver comprehensive cessation services to address nicotine addiction, combining behavioral interventions and smoking-cessation medications^10^. Evidence-based smoking cessation interventions demonstrate a clinically significant 2–15% increase in sustained tobacco abstinence rates (≥6 months) compared to no intervention controls^11^.

Having a quit intention is a prerequisite for preparing and taking action^12^. The lack of smoking cessation intention is one of the major challenges to quitting among Chinese smokers, and identifying the predictors is necessary for tobacco control^13^. Previous researchers have reported that the intention to quit smoking is associated with demographic factors, such as gender, ethnicity, income, and education^14-16^. Besides, social factors, such as health literacy and knowledge about smoking hazards, identity constructs, and self-efficacy were also influencing^17-19^.

Smokers who visit smoking cessation clinics have come to recognize smoking not merely as a habit but as a chronic addictive disorder requiring medical intervention^20^. These patients are often prompted to seek help by clear physical warnings, such as worsening cough or declining lung function, or by familial pressure^21^. In contrast, most regular smokers remain in a self-management phase, failing to acknowledge smoking as a genuine health threat fully. They frequently harbor cognitive biases, such as underestimating the cumulative damage smoking inflicts on the cardiovascular system or overestimating their perceived control over nicotine dependence^22^. While existing research has identified numerous factors associated with smoking cessation intention among current smokers, few studies have specifically examined these determinants among treatment-seeking smokers in smoking cessation clinics. This study utilized the questionnaire data of outpatients in standardized smoking cessation clinics in Ningbo City to characterize the demographic and smoking profiles of outpatients and further examine the possible association between nicotine dependence, motivations with the intention to quit smoking. The identification of these determinants enables healthcare providers to deliver precision smoking cessation interventions tailored to individual patient profiles.

## METHODS

### Study design and participants

We conducted a cross-sectional study of consecutive smokers who visited the standardized smoking cessation clinic in Ningbo City between January 2023 and January 2025. The inclusion criteria were current smokers (smoked daily for ≥12 months at the time of the survey), aged ≥18 years, motivated to quit or have already started cessation. The exclusion criteria were smokers who refused to participate in the survey. After the patients signed the informed consent form, they filled in the questionnaire on the web page of PAD. The questionnaires were completed by participants under the guidance of the smoking cessation clinic physicians.

### Measures

The design of the smoking cessation clinic questionnaire originated from the Chinese Center for Disease Control and Prevention (China CDC). The questionnaire comprises three sections: 1) Smoking-related characteristics: Including the validated Fagerström test for nicotine dependence (FTND), smoking history (duration), number of quit attempts, intention to quit, and motivations for cessation, etc; and 2) Demographic and health profile: gender, date of birth, marital status, education level, occupation, alcohol consumption patterns, and self-rated health status, etc. The following section provides detailed operational definitions for critical variables employed in this study.

### The Fagerström test for nicotine dependence

The Fagerström test for nicotine dependence (FTND) was used to assess the nicotine dependence of smokers. The score of the FTND ranges from 0 to 10, with higher scores indicating greater severity of dependence. Consistent with standard criteria, the nicotine dependence level was categorized by the scores as: mild (0–3), moderate (4–6), and severe (7–10)^23^.

### Motivations for quitting smoking

This item is derived from the question: ‘What was the primary reason for your decision to quit smoking this time?’. The motivations for smoking cessation can be categorized into four primary domains according to the question options: health-related, family-related, social-environmental, and self-management-related. Specifically, health-related motivations comprised three distinct dimensions, including self-disease factors, disease prevention, and the health of their families. Family-related motivations included three factors: 1) prepare for pregnancy; 2) don’t want kids to be influenced by smokers; and 3) family members advise quitting smoking. Society-related motivations were affected by the surrounding environment and restricted by the smoke-free policy. Self-management related motivation included being in control of life and improving teeth yellowing and bad odor caused by smoking.

### Intentions to quit smoking

This item is derived from the question: ‘When are you going to quit smoking?’. The response options for this question include: ‘I have already started quitting smoking’, ‘plan to quit today’, ‘within 7 days’, ‘within 30 days’, ‘within 6 months’, ‘after 6 months’, and ‘undecided’. While current smokers are generally considered to have high cessation intention if they plan to quit within 30 days, this study defines ‘high intention’ as smokers who plan to quit smoking within the next 7 days (including those already beginning abstinence) are considered to have a high intention to quit smoking, given that the participants are predominantly patients with pre-existing motivation to quit.

### Statistical analysis

SPSS Version 26.0 (IBM Corp, Armonk, NY, USA) was used for data analysis. Descriptive statistics were conducted to characterize the study population, with continuous variables expressed as means with standard deviations. Continuous variables such as age and duration of smoking were tested for normality using Shapiro-Wilk tests. Normally distributed continuous variables were analyzed with the independent samples t-test, while non-normally distributed continuous variables were analyzed with the Mann-Whitney U test. Categorical variables were presented as frequencies (n) and proportions (%) and compared using χ^2^ tests. Multivariable logistic regression analyses were conducted to assess associations between nicotine dependence (FTND score), motivation types, and quit intention, with adjustment for potential confounders, for which p<0.1 in the univariate analysis. The results are presented as adjusted odds ratios (AORs) with 95% confidence intervals (CIs). A two-tailed α level of 0.05 defined statistical significance.

## RESULTS

A total of 716 participants were initially assessed for eligibility, and 13 failed to complete the relevant questionnaires effectively, achieving a response rate of 98.18%. The study consisted of 703 smoking cessation outpatients, among whom 400 (56.90%) demonstrated strong quit intention. Demographic characteristics revealed predominantly married individuals (n=619; 88.05%), participants who had higher education (n=391; 55.62%), and those reporting monthly incomes of ≥7000 RMB (n=357; 50.79%). As presented in Table 1, significant disparities in sociodemographic characteristics emerged between high and low smoking cessation intention groups. Patients with a high intention to quit smoking were younger than the low intention group. Significant differences between groups were observed in both education level (χ^2^=16.43; p<0.05) and occupational distribution (χ^2^=31.70; p<0.001). Higher education level (college and above) was more prevalent in the high-intention group (33.50% vs 26.07%).

**Table 1:** Sociodemographic characteristics of smoking cessation outpatients in Ningbo, China, 2023–2025 (N=703)

*Characteristics*	*Overall* *(N=703)* *n (%)*	*High-intention group* *(N=400)* *n (%)*	*Low-intention group* *(N=303)* *n (%)*	*χ^2^*	*p*
**Age** (years), mean ± SD	50.29 ± 14.78	48.17 ± 14.42	53.09 ± 14.82	-	<0.001***
**Marital status**				3.07	0.22
Married	619 (88.05)	356 (89.00)	263 (86.80)		
Unmarried	79 (11.24)	43 (10.75)	36 (11.88)		
Divorced/separated/widowed	5 (0.71)	1 (0.25)	4 (1.32)		
**Education level**				16.43	0.001**
Primary school and lower	129 (18.35)	54 (13.50)	75 (24.75)		
Junior high school	183 (26.03)	103 (25.75)	80 (26.40)		
High school or other	178 (25.32)	109 (27.25)	69 (22.77)		
College and higher	213 (30.30)	134 (33.50)	79 (26.07)		
**Occupation**				31.70	<0.001***
Government institution staff	79 (11.24)	45 (11.25)	34 (11.22)		
Industrial worker	379 (53.91)	223 (55.75)	156 (51.49)		
Farmer	125 (17.78)	52 (13.00)	73 (24.09)		
Retired	59 (8.39)	29 (7.25)	30 (9.90)		
Other	61 (8.68)	51 (12.75)	10 (3.30)		
**Monthly income** (RMB)				6.96	0.07
<5000	71 (10.10)	38 (9.50)	33 (10.89)		
5000–7000	275 (39.12)	154 (38.50)	121 (39.93)		
7000–9000	159 (22.62)	81 (20.25)	78 (25.74)		
>9000	198 (28.17)	127 (31.75)	71 (23.43)		

Variable ‘age’ was analyzed with the independent samples t-test: t= -4.43.

* p<0.05.

** p<0.01.

*** p<0.001.

RMB: 1000 Chinese Renminbi about US$14.

As shown in Table 2, the duration of smoking among outpatients was 22.03 ± 13.60 years. FTND scores classified 45.09% (n=317) as mildly dependent, 42.96% (n=302) as moderately dependent, and 11.95% (n=84) as severely dependent. More than half of outpatients (57.75%) had attempted to quit smoking before. Patients with high intention to quit smoking had shorter smoking histories than the low intention group (20.09 ± 12.25 vs 24.58 ± 14.84; p<0.001). The high-intention patients reported a greater number of prior quit attempts (χ^2^=48.91; p<0.001). Alcohol consumption prevalence reached 70.27% across the study population. Notably, 61.31% of participants self-rated their health status as poor.

**Table 2:** Nicotine dependence scores and smoking-related characteristics of smoking cessation outpatients in Ningbo, China, 2023–2025 (N=703)

*Characteristics*	*Overall* *(N=703)* *n (%)*	*High-intention group* *(N=400)* *n (%)*	*Low-intention group* *(N=303)* *n (%)*	*χ^2^*	*p*
**Duration of smoking** (years), mean ± SD	22.03 ± 13.60	20.09 ± 12.25	24.58 ± 14.84	-	<0.001***
**Degree of nicotine dependence**				44.54	<0.001***
Mild	317 (45.09)	137 (34.25)	180 (59.41)		
Moderate	302 (42.96)	203 (50.75)	99 (32.67)		
Severe	84 (11.95)	60 (15.00)	24 (7.92)		
**Number of attempts to quit smoking**				48.91	<0.001***
Never tried	297 (42.25)	124 (31.00)	173 (57.10)		
1	156 (22.19)	102 (25.50)	54 (17.82)		
2–5	228 (32.43)	158 (39.50)	70 (23.10)		
≥6	22 (3.13)	16 (4.00)	6 (1.98)		
**Drinking**				4.75	0.029*
Yes	494 (70.27)	268 (67.00)	226 (74.59)		
No	209 (29.73)	132 (33.00)	77 (25.41)		
**Self-reported health**				27.88	<0.001***
Good	272 (38.69)	121 (30.25)	151 (49.83)		
Poor	431 (61.31)	279 (69.75)	152 (50.17)		

Variable ‘the duration of smoking’ was analyzed with the Mann-Whitney U test: U=4.285.

* p<0.05.

** p<0.01.

*** p<0.001.

There were differences in smoking cessation motivation patterns between the high-intention group and the low-intention group (Table 3). The proportion of patients in the high-intention group who quit smoking for health (χ^2^=213.03; p<0.001) and family (χ^2^=9.16; p<0.05) oriented motivations was significantly higher than that in the low-intention group. Conversely, social environmental motivations were reported less frequently among high-intention participants than in the low-intention group (χ^2^=5.34; p<0.05).

**Table 3:** Motivations to quit smoking of smoking cessation outpatients in Ningbo, China, 2023–2025 (N=703)

*Motivations to quit smoking*	*Overall* *(N=703)* *n (%)*	*High-intention group* *(N=400)* *n (%)*	*Low-intention group* *(N=303)* *n (%)*	*χ^2^*	*p*
**Health related motivation**				213.03	<0.001***
Illness	127 (18.07)	113 (28.25)	14 (4.62)	65.03	<0.001***
Take care of health and prevent diseases	307 (43.67)	245 (61.25)	62 (20.46)	116.60	<0.001***
For the health of family	309 (43.95)	196 (49.00)	113 (37.29)	9.59	0.002**
**Family related motivation**				9.16	0.003**
Prepare for pregnancy	6 (0.85)	4 (1.00)	2 (0.66)	0.24	0.70
Don’t want kids to be influenced by smokers	13 (1.85)	8 (2.00)	5 (1.65)	0.12	0.79
Family members advise quitting smoking	35 (4.98)	28 (7.00)	7 (2.31)	8.02	0.005**
**Society related motivation**				5.34	0.031*
Affected by the surrounding environment	24 (3.41)	6 (1.50)	18 (5.94)	10.31	0.003**
Restricted by smoke-free policy	5 (0.71)	4 (1.00)	1 (0.33)	1.10	0.397
**Self-management related motivation**				0.16	0.734
Be in control of life	69 (9.82)	32 (8.00)	37 (12.21)	3.45	0.073
Improve teeth yellowing and bad odor caused by smoking	31 (4.41)	23 (5.75)	8 (2.64)	3.96	0.062

* p<0.05.

** p<0.01.

*** p<0.001.

**Figure 1:**
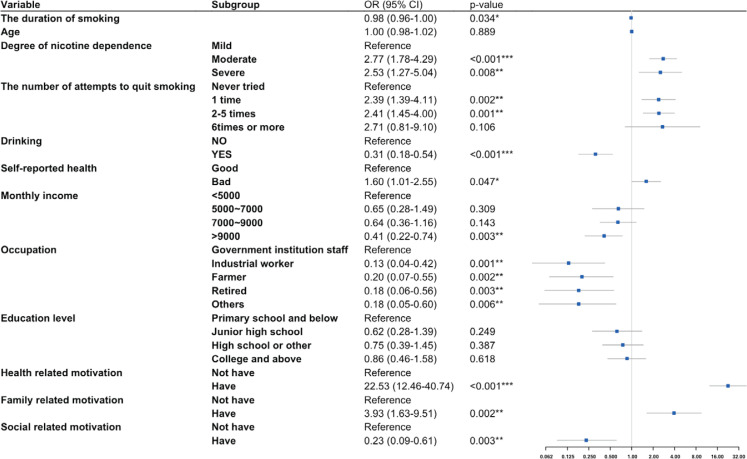
Factors associated with patients with the strong intention to quit smoking in Ningbo, China, 2023–2025 (N=703)

Logistic regression analyses of determinants of smoking cessation intention are presented in Table 4. The Hosmer-Lemeshow test showed no significant divergence between predicted and observed probabilities (χ^2^=3.20, df=8, p=0.92), indicating adequate model calibration. Neither age nor education level significantly influenced the intention to quit smoking. Compared to mild nicotine dependence, moderate (AOR=2.77; 95% CI: 1.78–4.29) and severe (AOR=2.53; 95% CI: 1.27–5.04) nicotine dependence groups were associated with significantly higher odds of strong cessation intention. Each additional year of smoking reduced the likelihood of high cessation intention by 2% (AOR=0.98; 95% CI: 0.96–1.00). The more attempts to quit smoking were associated with high intention to quit smoking, though this association diminished after six or more attempts. Those who drank were less likely to quit smoking than those who did not (AOR=0.31; 95% CI: 0.18– 0.54). Patients with poor self-rating status were more willing to quit smoking than those with good self-rating status (AOR=1.60; 95% CI: 1.01–2.55). Those with the highest monthly income (>9000 RMB) were less willing to quit smoking than those with the lowest monthly income (<5000 RMB). In the occupation category, industrial workers (AOR=0.13; 95% CI: 0.04–0.42), farmers (AOR=0.20; 95% CI: 0.07–0.55), and retired persons (AOR=0.18; 95% CI: 0.06–0.56) were significantly less willing to quit smoking than government institution staff (Figure 1).

**Table 4:** Factors associated with patients with the strong intention to quit smoking in Ningbo, China, 2023–2025 (N=703)

*Variables*	*β*	*SE*	*P*	*OR*	*95% CI*
**Degree of nicotine dependence** (control group: mild)					
Moderate	1.02	0.22	<0.001***	2.77	1.78–4.29
Severe	0.93	0.35	0.008**	2.53	1.27–5.04
**Duration of smoking**	-0.02	0.01	0.034*	0.98	0.96–1.00
**Age**	0.00	0.01	0.889	1	0.98–1.02
**Number of attempts to quit smoking** (control group: never tried)					
1	0.87	0.28	0.002**	2.39	1.39–4.11
2–5	0.88	0.26	0.001**	2.41	1.45–4.00
≥6	1.00	0.62	0.106	2.71	0.81–9.10
**Drinking** (control group: no)	-1.16	0.27	<0.001***	0.31	0.18–0.54
**Self-reported health** (control group: good)	0.47	0.24	0.047*	1.60	1.01–2.55
**Monthly income** (RMB) (control group: <5000)					
5000–7000	-0.43	0.43	0.309	0.65	0.28–1.49
7000–9000	-0.44	0.30	0.143	0.64	0.36–1.16
>9000	-0.90	0.31	0.003**	0.41	0.22–0.74
**Occupation** (control group: government institution staff)					
Industrial worker	-2.06	0.61	0.001**	0.13	0.04–0.42
Farmer	-1.62	0.52	0.002**	0.20	0.07–0.55
Retired	-1.70	0.57	0.003**	0.18	0.06–0.56
Other	-1.74	0.63	0.006**	0.18	0.05–0.60
**Education level** (control group: primary school and lower)					
Junior high school	-0.47	0.41	0.249	0.62	0.28–1.39
High school or other	-0.29	0.34	0.387	0.75	0.39–1.45
College and higher	-0.16	0.31	0.618	0.86	0.46–1.58
**Motivation** (control group: do not have)					
Health related	3.11	0.30	<0.001***	22.53	12.46–40.74
Family related	1.37	0.45	0.002**	3.93	1.63–9.51
Social related	-1.45	0.49	0.003**	0.23	0.09–0.61

* p<0.05.

** p<0.01.

*** p<0.001.

RMB: 1000 Chinese Renminbi about US$14.

## DISCUSSION

According to a 2018 national survey in China, only 16.1% of current smokers intended to quit smoking within the next 12 months^24^. Different from current smokers, smokers attending cessation clinics typically have higher intentions to quit smoking. The smoking cessation clinic outpatients in China mainly comprise two subgroups: 1) individuals actively seeking medical assistance for tobacco cessation, and 2) patients referred to the smoking cessation clinic due to smoking-related comorbidities or other health conditions, who typically require immediate smoking cessation for clinical reasons^25^.

Our study revealed a positive association between nicotine dependence and intention to quit smoking among smoking cessation outpatients. Specifically, patients with moderate and severe dependence (FTND ≥4) showed a higher willingness to quit smoking compared to those with mild dependence (FTND ≤3). In the outpatient setting, higher dependence may increase quit intention due to heightened awareness of health risks and a recognized need for medical assistance. However, among general smokers, greater dependence often reduces quit intention due to fear of withdrawal and lower confidence in their ability to quit independently. Highly dependent smokers at cessation clinics might represent a self-selected group with heightened health consciousness, whose perception of their addiction severity motivates them to seek professional help. In contrast, smokers with milder dependence might feel capable of quitting on their own. Previous evidence supports this interpretation, showing that smokers who were more aware of the health consequences of smoking exhibited stronger quit intentions^26^. Additionally, smoking cessation interventions should place greater emphasis on psychological assessments to deeply understand cessation intentions. Mansueto et al.^27^ developed the Smoking Abstinence Expectancies Questionnaire (SAEQ), which has four subscales: negative mood, somatic symptoms, harmful consequences, and positive consequences. It can be used at smoking cessation clinics to discriminate between subjects with different levels of smoking abstinence expectancies^27^. The study of Fan et al.^28^ on chronic obstructive pulmonary disease (COPD) patients further revealed that patients with more severe psychological anxiety tend to be more willing to quit smoking. Both studies highlight the role of psychological assessments in understanding the intention to quit smoking.

Our study identified differences in the types of motivations among smoking cessation clinic outpatients. The high-intention group was more health- and family-driven, while the low-intention group was more dependent on society and self-management. This distinction helps explain why parents who acknowledge smoking-related health risks or benefits for their children tend to have stronger motivation to quit^29^. In this study, self-reported poor health status significantly increased willingness to quit, as most patients in poor health already recognize the severity of smoking-induced disease progression. For smokers with smoking-related cancers, perceived cancer-related benefits of quitting are correlated with quit intentions. Therefore, future clinical interventions should reinforce motivation through tailored strategies: for high cessation intention patients, provide medical evidence (e.g. cessation’s impact on disease management) and strengthen family-oriented incentives (e.g. protecting children from secondhand smoke)^30^. For low cessation intention patients, leverage social support networks (e.g. quit-smoking groups, peer encouragement) to address self-management barriers.

Consistent with other studies, smoking duration showed an inverse relationship with quit intention^31^. Alcohol consumption was negatively correlated with smoking cessation intention, which aligns with tobacco-alcohol co-use patterns^32^. Alcohol drinking has been consistently identified as a predictor of reduced smoking cessation success^33^. The more times attempting to quit smoking were associated with strong smoking cessation intention, consistent with prior research^34^. They were eager to quit smoking but had not been able to quit on their own, so they came to the smoking cessation clinic for medical help.

### Limitations

First, we acknowledge the potential for residual confounding as key variables such as cognition of the harm of smoking and psychological factors were not captured in our dataset. Second, this study had a limited representation of female participants, highlighting the need for future research to prioritize expanding sample diversity through large-scale, population-based epidemiological investigations. Third, it may also be influenced by recall bias because of self-report. Due to its cross-sectional design, temporality is unknown, and causal relationships cannot be claimed. Moreover, it is important to consider potential volunteer bias arising from instances where several participants may demonstrate significant non-compliance by refusing to complete the assessment instruments. Finally, the binary categorization of smoking cessation intention (high vs low) may lead to misclassification bias. The classification of motivation for quitting smoking is rather subjective, but as reasonable as possible.

## CONCLUSIONS

The intention to quit smoking among smoking cessation outpatients was positively associated with the degree of nicotine dependence. Outpatients with health- and family-oriented motivations were more willing to quit smoking. Doctors should take these characteristics into account to provide personalized smoking cessation services.

## Data Availability

The data supporting this research cannot be made available for privacy or other reasons.
